# Pelvic organ prolapse surgical training program in Bangladesh and Nepal improves objective patient outcomes

**DOI:** 10.1007/s00192-020-04562-4

**Published:** 2020-10-14

**Authors:** Barbara Hall, Judith Goh, Maqsudul Islam, Anubha Rawat

**Affiliations:** 1 DAK Foundation, Sydney, Australia; 2 Greenslopes Private Hospital, Brisbane, Australia; 3 Gold Coast University Hospital, Gold Coast, Australia; 4 Griffith University School of Medicine, Gold Coast, Australia; 5 QE2 Hospital, Brisbane, Australia; 6 Griffith University, Gold Coast, Australia; 7 Impact Foundation, Dhaka, Bangladesh

**Keywords:** Low-resource settings, Pelvic organ prolapse, Sacrospinous fixation, Surgical training, Surgical outcomes, Vaginal repair

## Abstract

**Introduction and hypothesis:**

The DAK Foundation (Sydney) has facilitated pelvic organ prolapse (POP) repairs performed by local gynecologists for underprivileged women in Bangladesh and Nepal since 2014. Initially, there was no long-term patient follow-up. When 156 patients were examined at least 6 months after their surgery, an unacceptably high rate of prolapse recurrence and shortened vaginas was identified. This demonstrated the need for surgical up-skilling in both countries. Our hypothesis is that the introduction of a surgical training program in low-resource countries can significantly improve patient outcomes after pelvic floor surgery.

**Methods:**

One-on-one surgical re-training was undertaken to up-skill the gynecologists in fascial vaginal repair and vaginal apical reconstruction utilizing sacrospinous fixation (SSF). Following the surgical up-skilling, a further 289 women (between 6 and 18 months post-operatively) were examined to determine patient outcomes. Outcome measures were:

1. Prolapse recurrence: POPQ (pelvic organ prolapse quantification [1]) ≥ stage 2
2. Vaginal length < 4 cm

**Results:**

Prior to implementation of the surgical training program, 76% of patients had recurrent prolapse ≥ stage 2, and 56% had a vagina < 4 cm in length. Following the training program, prolapse recurrence was reduced to 45% with significant reductions in the apical, anterior and posterior compartments. The incidence of unacceptable vaginal shortening was 4%. We could not rely on patient symptoms to determine whether they had recurrences.

**Conclusion:**

Clinical patient follow-up to determine surgical outcome is essential in low-resource settings. We have demonstrated that surgical up-skilling in vaginal hysterectomy, vaginal repair and introduction of SSF were necessary to achieve acceptable prolapse recurrence rates in our programs in Bangladesh and Nepal.

## Introduction

Pelvic organ prolapse (POP) has a profound impact on the quality of life of women—especially women in low-resource countries. Limited evidence currently exists regarding the rate of POP in such women; however, the rate of major prolapse seems to be higher than in higher resource countries. This is probably due to differences in lifestyle, nutrition and parity.

A 2016 study of women in rural Bangladesh showed a 15.6% prevalence of this condition [1] according to data provided by questionnaires relating to symptoms. The 2016 Bangladesh Maternal Mortality and Health Care Survey [2] documented a self-reported prevalence of any stage of POP of 23.5% but the Maternal Morbidity Validation Survey [3] demonstrated considerable over-reporting of symptoms, predicting an adjusted prevalence of pelvic organ prolapse quantification (POPQ) [4] stage 3 or 4 prolapse in 11.4 per 1000 Bangladeshi women who have ever given birth. This suggests that there are more than 535,000 women in Bangladesh living with major POP. Half of these women are aged between 15 and 49 years.

The prevalence of POP varies greatly across Nepal, but a 2006 UNFPA study [5] across rural and urban districts showed a prevalence of 10.4% (any stage), while a 2016 Nepal government study [6] reported that 6.4% of women of reproductive age (15 to 49 years) suffer from POP (stages not specified).

In 2014, the DAK Foundation (Sydney) alongside the Impact Foundation Bangladesh implemented a program for POP surgery for under-privileged women in 15 private centers (mostly rural) across Bangladesh. From January 2014 to December 2019, 10,471 women underwent surgery. Two hundred fifty-six surgeries have also been completed in one private urban center in Nepal (Kathmandu) since 2016. The surgeons in the program in both countries are general gynecologists, as there was, at the time, no Urogynecology subspeciality program in either country. Their clinics receive financial compensation; however, there is no cost to the patient. With consent, detailed data have been collected from each of these patients, including demographic details, clinical staging and type of operation performed.

Surgical intervention is a frequently used treatment option for major prolapse; however, in most low-resource settings there are no data on postoperative prolapse recurrence rates. This is because patients are generally unable to attend for follow-up for social, economic and logistic reasons. Without adequate follow-up, it is impossible to assess the efficacy of surgical intervention. Because of this, the DAK and Impact foundations instituted postoperative review of their program, beginning in August 2015, with the hypothesis that surgical upskilling might be necessary. The findings indeed demonstrated the need for surgical up-skilling, leading to the development and implementation of a surgical training program for all the gynecologists. Having identified the need for up-skilling, the hypothesis of this study is that a surgical training program for pelvic organ prolapse in low-resource settings can improve patient outcomes. We aim to demonstrate this by comparing patient outcomes before and after the implementation of the training program. We have used prolapse recurrence rates (POPQ stage ≥ 2) and vaginal length (< 4 cm) as our indicators of patient outcomes. The aim of surgical up-skilling is to achieve prolapse recurrence rates comparable to those reported in the current literature [7] and vaginas of normal length.

## Materials and methods

In August 2015, the DAK Foundation began to audit surgeries and outcomes. Two Australian gynecologists (BH, JG) who had not been involved in the initial program started reviewing individual surgeon’s techniques and performed clinical examinations on 156 women who had undergone ‘classic pelvic floor repair’ within the program. In both Bangladesh and Nepal, this meant vaginal hysterectomy with anterior repair (without fascial plication), infrequent posterior repair and rarely perineorrhaphy. The mean postoperation period for reviewed patients was 12 months; their surgery was for POPQ stage 3 or 4 prolapse and did not include an apical support procedure.

The up-skilling program commenced in December 2015. Three of the authors (BH, AR, MI) traveled to each region over the subsequent 3 years, providing program and administration support, and conducted theoretical and surgical hands-on training in vaginal hysterectomy, anterior and posterior repair with fascial plication, modified McCall culdoplasty (following vaginal hysterectomy) and sacrospinous fixation (SSF). Apart from vaginal hysterectomy, none of these procedures had been part of the routine classic pelvic floor repair, performed by the surgeons previously. Training in high uterosacral suspension was not part of this program. One of the surgeons was already skilled in this procedure and preferred it for some patients. Some of the surgeons were already competent in colpocleisis. Training in the standard colpocleisis procedure was provided for those who had not previously performed it if a patient suitable for the procedure presented during the training period.

The theoretical training involved lectures on all aspects of prolapse—etiology, classification and management. Supplementary written material was also provided. We introduced POPQ staging [4] to standardize documentation for data collection. To date, 17 doctors have been trained in private clinics in Bangladesh and 4 in Nepal. Training has also been provided in two government hospitals in Bangladesh—Dhaka Medical College Hospital and Sylhet Medical College Hospital—and in one government hospital in Nepal, Paropakar Maternity and Women’s Hospital Thapathali. The average length of hospital stay was 5 days; we encouraged this as the majority of patients lived in rural communities a considerable distance from the hospital, potentially making a return to the hospital very difficult if any complications arose. Many would also have had little opportunity for rest at home due to home duties and farming work.

Polydioxanone (PDS) sutures (1 PDS for the SSF and 2-0 PDS for fascial repair) were introduced because of data demonstrating a statistically significant reduction in anterior compartment recurrence when PDS was used rather than polyglactin for the fascial repair [8]. Each surgeon was provided with the PDS sutures (they were not readily available in either country) in addition to a left handed Deschamp aneurysm needle for right-sided SSF, a Briesky retractor and a suture-retrieving hook. After receiving 3 to 4 days of individual training, including supervision in performing at least 12 POP repair cases with SSF, each surgeon then continued to perform up to 20 procedures per month as part of the program.

The same trainer (BH) re-visited each surgeon 8 to 12 months after training to reassess surgical technique, give additional training as required and clinically assess postoperative results by patient examination. Post-training, postoperative outcome data were collected for 289 patients who were selected on the basis of their ability, and willingness, to attend for clinical examination.

Each private center, government hospital, surgeon and patient signed a Memorandum of Understanding, giving ethical and legal consent for surgical training, clinical patient follow-up and patient data collection for research purposes. Ethics approval was also obtained from the Greenslopes Hospital (Brisbane) Ethics Committee (Ethics Approval Protocol 18/19). The study has been registered with the Australian New Zealand Clinical Trials Registry (ACTRN) Number 1261800502235.

The results of the study were collated and exported to the statistical package for the social sciences (SSPS) for data analysis. The two proportion test (chi-square test) and first principle manual calculations were performed. Manual calculations were also used for the standard deviation data.

## Results

Table 1 shows demographic data for the total 10,471 Bangladeshi patients who were operated on between 2014 and December 2019. The frequencies of their most common presenting symptoms are presented in Table 2. While 74% of patients had difficulty passing urine, and 30% had urinary incontinence, we did not include analysis of voiding dysfunction in this study. Urodynamics is not available in these centers, and we were unable to determine the etiology of voiding dysfunction from the questionnaires.

**Table 1:** Demographic data. (10,471 Bangladeshi patients)

	Mean	SD
Age (years)	53.6	10.6
Height (cm)	149.4	6.9
Weight (kg)	48.4	7.6
BMI	21.7	
Parity	4.6	2.2
Age at first delivery (years)	16.9	1.8
Patients having first delivery by 20 years age	98%	
Deliveries at home	99%	
History of difficult or obstructed labor	9.5%	
History of at least one cesarean section	0.1%	
Family history of prolapse	5.5%	
Previous hysterectomy (for prolapse or other condition)	2%	
Post-menopausal	74%	
Married	78%	
Sexually active	57%

**Table 2:** Most common presenting symptoms

Feeling of a vaginal bulge	99.8%
Average length of time bulge present (years)	7.6
Vaginal pain or discomfort	36%
Difficulty passing urine	74%
Urinary incontinence	30%
Difficulty evacuating bowel	28%

Among the pre-training follow-up cohort of 156 patients, the overall rate of recurrent POP (≥ stage 2) was 76% with a standard deviation of 16.9%. Within this group, 52% (SD 26.2%) had apical recurrence, 39% (SD 29.9%) had anterior recurrence, and 31% (SD 16.6%) had posterior recurrence. In 56% (SD 32.0%) of patients the vagina was < 4 cm in length.

The reason for the high rates of prolapse recurrence and shortened vaginas was determined by observation of surgical techniques, and there was little variation in technique across both countries. Wide dissection and removal of excess vaginal skin lateral to the cervix accounted for the gross vaginal shortening, while lack of apical suspension was responsible for the high apical recurrence rate. Inadequate bladder and bowel dissection and lack of fascial plication resulted in the high anterior and posterior recurrence rates. We do not have data on complication rates for the pre-training cohort of patients.

Between December 2015 and December 2019, 6890 patients had surgery performed by up-skilled surgeons. All patients had stage 3 or 4 prolapse, with preoperative data showing the majority (64%) of these patients had stage 3 prolapse, while the remainder (36%) had stage 4. Anterior compartment prolapse was present in 99%, posterior compartment prolapse in 94% and apical prolapse in 97%. Operative data showed that 93% of patients had a vaginal hysterectomy, 99% had an anterior repair, 95% had a posterior repair, 74% had a perineorrhaphy, 80% had a sacrospinous colpopexy, 4% had a sacrospinous hysteropexy, and 2% had a high uterosacral suspension. Colpocleisis was chosen for 1% patients because of symptomatic stage 4 prolapse with associated severe medical comorbidities or advanced age. Each of these patients had failed a trial of vaginal pessary.

**Fig. 1:**
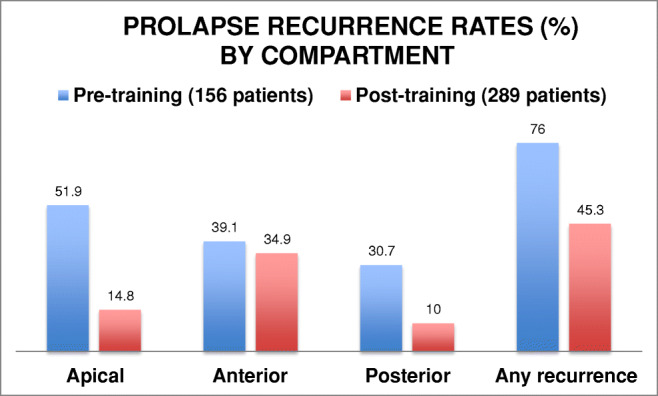
Prolapse recurrence rates by compartment

Between November 2016 and December 2019, 289 patients whose surgeon had received up-skilling training attended for clinical review. They were all between 6 and 12 months post surgery. The overall prolapse recurrence rate was 45% (SD 16.4%), representing a statistically significant reduction (from 76%, *p* < 0.00001) by surgical up-skilling and the addition of SSF. The apical recurrence rate was reduced from 52% to 15% (SD 10.7%), which is also highly significant, *p* < 0.00001, as was the reduction in the posterior compartment recurrences–31% reduced to 10% (SD 10.2%) with *p* < 0.00001. There was a reduction in anterior compartment recurrence, from 39% to 35% (SD 18.8%), but this did not achieve statistical significance. These results are compared in Fig. 1.

**Fig. 2:**
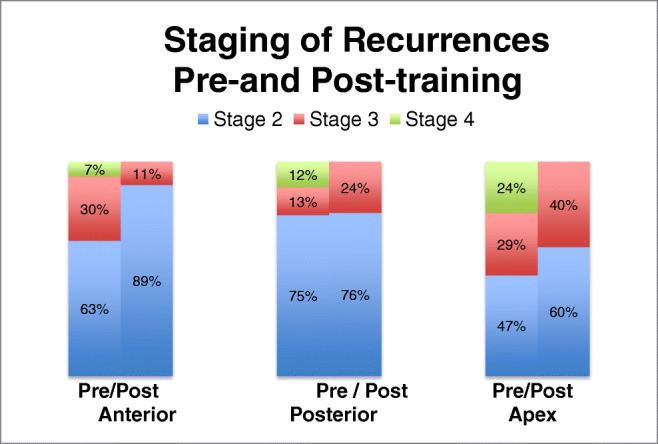
Comparison of the staging of postoperative recurrences after surgery performed before and after the training

The majority of all compartment recurrences were stage 2: 60 % of apical recurrences, 89% of anterior recurrences and 76% of posterior recurrences being stage 2. The remainder of the recurrent prolapses were stage 3 with no patient having a stage 4 recurrence. Figure 2 compares the staging of postoperative recurrences after surgery performed before and after the training and shows a marked reduction in the percentage of stage 3 and 4 recurrences in each compartment.

The second aim of the study was to achieve functionally normal vaginas. Pre-training incidence of unacceptably short vaginas (< 4 cm) was 56% (SD 32.0%), and post-training this was significantly reduced to 4% (SD 7.2%), *p* < 0.00001.

The vast majority of these postoperative review patients were asymptomatic and only admitted to symptoms when specifically asked. In fact, only 33% of those patients with stage 3 or 4 recurrence admitted to being aware of a vaginal bulge.

Despite our attempts to obtain complete data, only one surgeon has consistently reported surgical complications, so a full analysis of complication rates for the whole cohort is not possible. Analysis of the last 219 cases from this surgeon (Table 3) shows rates consistent with those reported in the literature.(7).

**Table 3:** Surgical complications. (219 patients from one surgeon)

Vault infection	5%
Urinary retention	8.2%
Urinary tract infection	6.8%
Buttock pain	7.8%
Hemorrhage (unspecified)	5%

## Discussion

After up-skilling, the prolapse recurrence rates in each compartment are within the ranges reported in a large systematic review published by Tseng et al. in 2013 [7]. This has fulfilled the first aim of the study. The standard variations attributable to the prolapse recurrence rates indicate a wide variation between individual surgeons; however, this variation is less in the post-training than in the pre-training recurrence results.

SSF was chosen as the best procedure for vault support for this program as it is least invasive, has a short recovery time and is least expensive for low-cost hospital settings. SSF results in a lower apical recurrence rate than most other per-vaginal vault support procedures [9] and has comparable complication rates. Reported complications include hemorrhage, rectal injury, buttock pain, sciatic nerve damage, vault infection, urinary tract infection, urinary retention and ureteric injury. The ureteric injury rate is lower than that for high uterosacral ligament suspension [10], which is an important consideration in low-cost hospital settings where routine cystoscopy is not available.

Many of the reviewed post-training surgeries were performed relatively early in the surgeon’s learning curve, and the results presented are the composite of 17 individual surgeons, with varying degrees of competence with the SSF procedure—as indicated by the wide standard deviations on the recurrence data. Post-training data show a total apical suspension rate (other than McCall culdoplasty) of 86%. This should increase to 100% as surgeon competence and confidence increases, causing a further reduction in the apical prolapse recurrence rate. Post-training follow-up visits by the trainer are invaluable, not only to assess patient outcomes, but also to reinforce the usefulness of the new techniques and provide additional top-up training. The SSF procedure does have a steep learning curve, but once mastered is a quick and relatively safe procedure.

The recurrence rate of anterior compartment prolapse in this study is disappointing—but still within the ranges reported in the literature [7]. It is accepted that SSF predisposes to anterior compartment recurrence because of posterior angulation of the vagina, so that the high rate (35%) of anterior recurrences could, in part, be explained by the high percentage (84%) of concomitant SSF in this cohort. During post-training reassessment of the surgeons’ techniques, however, we noted that many still required some further training in anterior colporrhaphy. Their dissection of the bladder from the vaginal skin was often still inadequate, because they feared the patient would develop postoperative urinary retention with more extensive dissection. With the provision of further training, we are hopeful that the incidence of anterior compartment recurrence will decrease.

When initially questioned, the surgeons did not consider that a shortened vagina was a problem as they felt that the majority of patients were ‘older’ and widowed. They were surprised to find that the data demonstrated that only 18% of patients were > 65 years, 78% were married and 57% were sexually active prior to the surgery. Many others were not sexually active because of the prolapse. Thus, postoperative functional vaginal length is an important factor, as the majority of patients were, or would like to be, sexually active.

Despite the fact that all postoperative review patients had initially presented with symptomatic stage 3 or 4 prolapses, only 33% of those with clinically demonstrated stage 3 and 4 postoperative prolapse recurrence admitted to symptoms—and the vast majority only on direct questioning. The reason for this is uncertain. It does indicate, however, that clinical examination, rather than administration of a symptom questionnaire, is essential to determine adequacy of the surgical outcome in these populations. It is also important to note that these patients only presented for follow-up when actively encouraged to do so. Even the symptomatic patients did not voluntarily present requesting assessment or treatment. This emphasizes the importance of clinical follow-up in low-resource settings where most surgeons are unaware of the high rate of postoperative prolapse recurrence.

This study is focused on the surgical management of major prolapse. At the time of implementing the program, conservative management with pessaries was rarely employed because only hard rubber pessaries were available in each country. These were associated with unacceptable complications. In mid 2018, we introduced to Bangladesh soft silicone pessaries manufactured in Australia by the medical device company Gynaecologic. All of the surgeons on our program have been provided with pessaries, and they and their nurses have been trained in insertion and management. We are hopeful that we will be able to present data on pessary usage in the future.

There are two major limitations to this study. First, follow-up is only short term (6 to 18 months). Recurrence rates usually increase with time; however, we hope that this will be offset by a further reduction in recurrence rates with increasing surgeon experience and confidence with the SSF procedure, so that closer to 100% of patients will have an apical suspension procedure performed. Our aim is to obtain long-term follow-up as the program continues.

The second limitation is our lack of presentation of complication rates. Collected data from one surgeon (and anecdotal reports from the others) suggest rates comparable with reports in the current literature; however, at this point we have been unable to collect complete data from all surgeons. This is a major aim for the ongoing program.

## Conclusions

Improved vaginal hysterectomy technique, fascial vaginal repair and SSF have been demonstrated to lead to reduction in prolapse recurrence and unacceptably shortened vaginas. Assistance with surgical teaching is invaluable for gynecologists working in low-resource settings, as individual hands-on training is often limited. Our aim in both Bangladesh and Nepal is to train local gynecologists who will eventually become master trainers for their colleagues, improving country-wide patient outcomes. Patient follow-up and clinical examination (rather than administration of symptom questionnaires) are essential to accurately assess patient outcomes of POP surgery in low-resource settings, as many patients are unwilling to admit to symptoms of prolapse recurrence postoperatively. Continuing long-term follow-up and complete collection and analysis of complications are our aims for the next phase of this program.

## References

[1] Akter F, Gartoulla P, Oldroyd J, Islam R (2016). Prevalence of, and risk factors for, symptomatic pelvic organ prolapse in rural Bangladesh: a cross-sectional survey study. Int Urogynecol J.

[2] Bangladesh Maternal Mortality and Health Care Survey. 2016. [4] Chowdhury S. Editorial Bangladesh J Obstet Gynaecol 2010;25(1):1-2.

[3] Maternal Morbidity Validation Survey 2016. Prevalence of obstetric fistula and pelvic organ prolapse in Bangladesh: summary of the 2016 National Estimates. MEASURE evaluation/USAID.

[4] Bump RC, Mattiasson A, Bo K, Brubaker LP, DeLancey JO, Klarskov P, Shull BL, Smith AR (1996). The standardization of terminology of female pelvic organ prolapse and pelvic floor dysfunction. Am J Obstet Gynecol.

[5] Unite Nations Population Fund (UNFPA). Status of reproductive morbidities in Nepal. 2006 Accessed on June 2016. Available at: http://un.org.np/sites/default/files/report/tid_67/2009-03-17-UNFPA-status-morbidity.pdf

[6] United Nations Population Fund. Government of Nepal. Ministry of Health. Family Health Division. Study on selected reproductive health morbidities among women attending reproductive health camps in Nepal. 2016. Accessed on Aug 2018. Available at: https://nepal.unfpa.org/sites/default/files/pub-pdf/RH%20Morbidity%20study_0.pdf.

[7] Tseng L, Cheng I, Chang S, Lee C (2013). Modern role of sacrospinous ligament fixation for pelvic organ prolapse surgery - a systemic review. Taiwan J Obstet Gynecol.

[8] Bergman I, Westergren Soderberg M, Kjaeldgaard A, Ek M (2016). Does the choice of suture material matter in anterior and posterior colporrhaphy?. Int Urogynecol J.

[9] Barber MD, Maher C (2013). Apical Prolapse. Int Urogynecol J.

[10] Karram M, Walsh P (2001). High uterosacral vaginal vault suspension with fascial reconstruction for vaginal repair of enterocoele and vaginal vault prolapse. Am J Obstet Gynaecol.

